# Moderate-Intensity Aerobic Exercise Restores Appetite and Prefrontal Brain Activity to Images of Food Among Persons Dependent on Methamphetamine: A Functional Near-Infrared Spectroscopy Study

**DOI:** 10.3389/fnhum.2019.00400

**Published:** 2019-11-13

**Authors:** Hongbiao Wang, Yifan Chen, Xiawen Li, Jiakuan Wang, Yu Zhou, Chenglin Zhou

**Affiliations:** ^1^Department of Physical Education, Shanghai University of Medicine & Health Sciences, Shanghai, China; ^2^Department of Sport Psychology, School of Sport Science, Shanghai University of Sport, Shanghai, China

**Keywords:** aerobic exercise, food reward, fNIRS, drug dependence, methamphetamine

## Abstract

The brain prefrontal control system is critical to successful recovery from substance use disorders, and the prefrontal cortex (PFC) regulates striatal reward-related processes. Substance-dependent individuals exhibit an increased response to drug rewards and decreased response to natural, nondrug rewards. Short-term aerobic exercise can ameliorate craving and inhibitory deficits in methamphetamine users, but the effect of exercise on food reward is unknown. This study used functional near-infrared spectroscopy (fNIRS) to measure the effects of moderate- and high-intensity short-term aerobic exercise on prefrontal activity related to food images and recorded the subjective feelings of appetite in methamphetamine-dependent users. In total, 56 men who met the *Diagnostic and Statistical Manual of Mental Disorders (Fifth Edition)* criteria for methamphetamine dependence, with a mean (SD) body mass index of 24.7 (3.5) kg/m^2^ and age of 30.2 (5.1) years, were randomly assigned to one of two exercise groups: moderate intensity (*n* = 28; 65%–75% of maximum heart rate) and high intensity (*n* = 28; 76%–85% of heart rate maximum). Each group also performed a resting control session for 35 min 1 week before or after the exercise, in a counterbalanced order. Mean oxygenated hemoglobin concentration changes in the PFC when viewing visual food cues were assessed by fNIRS, and subjective feelings of appetite were self-rated using visual analog scales after moderate- or high-intensity aerobic exercise and after the resting control session. A continuous-wave NIRS device was used to obtain functional data: eight sources and seven detectors were placed on the scalp covering the PFC, resulting in 20 channels per participant. We found that moderate-intensity aerobic exercise significantly increased both, the activation of the left orbitofrontal cortex (OFC) to images of high-calorie food (*P* = 0.02) and subjective sensations of hunger (*F*_(1,54)_ = 7.16, *P* = 0.01). To our knowledge, this study provides the first evidence that moderate-intensity aerobic exercise increases OFC activity associated with high-calorie food images and stimulates appetite in methamphetamine-dependent individuals. These changes suggest that exercise may reestablish the food reward pathway hijacked by drugs and restore sensitivity to natural rewards. This evidence may contribute to the development of specific exercise programs for populations with methamphetamine dependence.

## Introduction

Drug addiction is considered a chronic brain disease (Volkow and Morales, 2015). Long-term use of addictive substances leads to lasting changes in the brain structure and function of individuals, including the reward system, which is considered the basis for the development and maintenance of substance addiction (Robinson and Berridge, 1993; Koob and Volkow, 2010; Noël et al., 2013). Methamphetamine (MA) is the second most common illegally used drug in the world, and no drugs have shown efficacy in treating MA dependence (Rawson, 2013). Long-term MA use has been linked to repeated relapse episodes, possibly exacerbated by cognitive impairment during drug withdrawal (Dean et al., 2013; Bernheim et al., 2016). Despite our increased understanding of MA, the effects of repeated MA use, and the severity of the problem, no treatment has been consistently effective in alleviating the symptoms of MA addiction, especially in terms of cognitive impairment and drug-seeking (O’Brien, 2005; Brackins et al., 2011).

Therefore, measures must be taken to diminish cognitive impairment during drug withdrawal. Cognitive impairment, especially within the reward system, in users dependent on drugs continues even after withdrawal has begun. Several studies have reported a decrease in dopamine D2 receptors and dopamine release in the striatum of individuals dependent on drugs that can persist for months after detoxification (Gradin et al., 2014). This finding has been reported for various addictive drugs, including cocaine, alcohol, methamphetamine, and nicotine (Wang et al., 1997; Fehr et al., 2008; Volkow et al., 2009). These persistent neuroadaptive changes may lead to reduced sensitivity to nondrug reinforcers (Koob and Volkow, 2010) and may even impair the ability to respond adequately to rewards unrelated to the drug even during abstinence (Wrase et al., 2007). Individuals dependent on substances show a decreased response in the striatum to natural rewards and thus appear to search for alternative stimuli (drugs) to maintain their equilibrium (Garavan et al., 2000; Koob and Le Moal, 2001; Paulus et al., 2005; Lubman et al., 2009; Volkow et al., 2009, 2010). Brain regions associated with substance abuse and natural reward overlap (Garavan et al., 2000; Karama et al., 2002), for example, with the food reward region (DiLeone et al., 2012), which supports the hypothesis that drug dependence “hijacks” the natural reward pathways, leading to overestimation of drug-related rewards and underestimation of nondrug-related rewards (Diekhof et al., 2008; Feltenstein and See, 2008).

It has been reported that MA-dependent research participants consume large amounts of food during at least the first month of abstinence (Zorick et al., 2012), suggesting that in the absence of the ability to choose MA, appetite for food is not impaired. A preclinical drug vs. food choice procedure has been used to evaluate candidate medications for MA use disorder (Banks, 2017), indicating that intervention must be applied after abstinence to restore appetite for food. Thus, food reward, as a natural reward, has been used to evaluate the sensitivity to natural reward vs. drug reward after abstinence among individuals who use drugs. The region of the brain that responds to a natural reward should be reclaimed from responding to drugs by using interventions to increase brain activation in the appropriate region of individuals dependent on drugs in response to that natural reward stimulus, such as food.

Aerobic exercise may be a substance use disorder treatment method (Pareja-Galeano et al., 2013; Wang et al., 2014; Linke and Ussher, 2015). Moderate-intensity short-term aerobic exercise was found to reduce drug craving in MA-dependent individuals and to promote recognition of normal and drug-related inhibitory control (Wang et al., 2015, 2016). Recent functional neuroimaging findings also suggest that long-term regular exercise may alter how brain reward regions respond to visual food cues (Cornier et al., 2012; Nock et al., 2012), and short-term aerobic exercise may change the neuronal responses in food reward brain regions, regardless of whether that aerobic exercise is moderately intense (Crabtree et al., 2014) or of high intensity (Evero et al., 2012).

The prefrontal cortex (PFC) regulates striatal reward-related processes and exhibits activity that predicts treatment outcome with respect to maintaining abstinence (Garavan and Weierstall, 2012). Neuroscience research has pointed to the importance of the orbitofrontal cortex (OFC) in food-seeking because of its responsiveness to changes in the reward value of stimuli (Thorpe et al., 1983; Rolls, 1984, 1990). The use of functional near-infrared spectroscopy (fNIRS) technology has been emerging in the field of cognitive neuroscience in recent years. This technology can be used to directly reflect the hemodynamic changes in cerebral cortical areas, including the PFC. Functional magnetic resonance imaging (fMRI) and fNIRS have a common neurophysiological basis, namely, the neurovascular coupling mechanism. NIRS is an optical technique that noninvasively measures changes in hemoglobin and oxygenation in the human brain (Jöbsis, 1977).

The working principle of NIRS is that neural activity in a brain region results in increased glucose and oxygen consumption at local capillary beds. The increased cerebral blood flow carries oxygen to the active areas, and this temporarily exceeds local neuronal oxygen utilization, resulting in an overabundance of cerebral blood oxygenation in active areas. Thus, NIRS can be used as an index of neural activity. Changes in cerebral blood flow and oxygenation are closely related to neural activity. Changes in oxygen-containing hemoglobin concentrations during the task reflect neuronal activity because they are associated with induced changes in regional cerebral blood flow (Hock et al., 1995; Tanida et al., 2004; Irani et al., 2007). When neurons become active, local blood flow to related brain regions increases, and oxygenated blood replaces deoxygenated blood. Among the three NIRS parameters (oxyHb, deoxyHb, and totalHb; Hoshi et al., 2001; Strangman et al., 2002), the change in oxyHb is the most sensitive indicator of regional cerebral blood flow change; thus, the measured oxyHb concentration can be used to directly reflect hemodynamic changes. Although the spatial resolution of NIRS is lower than that of other functional neuroimaging methods, such as positron emission tomography and fMRI, NIRS has the advantages of high time resolution (<0.01 s) and that it can be performed under natural conditions (Miyai et al., 2001). Thus, NIRS is arguably the best choice for use at the drug rehabilitation bureau.

The objective of the current study was to investigate the short-term effects of moderate- and high-intensity aerobic exercise training on prefrontal activity related to images of high-calorie and low-calorie foods among individuals who were MA-dependent. Subjective sensations of hunger, fullness, and desire to eat were also self-reported after exercise to examine differences in appetite. On the basis of the current literature, we hypothesized that there would be a dose-response effect of exercise intensity leading to increased prefrontal activity to high-calorie foods images relative to that in a non-exercise control condition among MA-dependent individuals.

## Materials and Methods

### Ethical Statement and Study Participants

This study was conducted according to the guidelines laid down in the Declaration of Helsinki and was approved by the ethics committee of Shanghai University of Sport (No. 102772019RT041). Written informed consent was obtained from all participants before enrolling them in the study.

In total, 56 men [mean and standard deviation (SD) age, 30.2 (5.1) years; mean (SD) body mass index (BMI), 24.7 (3.5) kg/m^2^] who met the Diagnostic and Statistical Manual of Mental Disorders (Fifth Edition) criteria for MA dependence were recruited from the Drug Rehabilitation Bureau of Shi Liping in Zhejiang province. Participants were included if they met the following criteria: (1) no metabolic or chronic disease, no medical conditions, and not taking medication known to influence gastric emptying or appetite; (2) no eating disorder, psychiatric diagnoses, or neurological illness; (3) weight stable (<3 kg change in the last 3 months); (4) aged 18–45 years; (5) right-hand dominant; and (6) abstinent and receiving treatment for 3 months.

### Study Design

**Figure 1:**
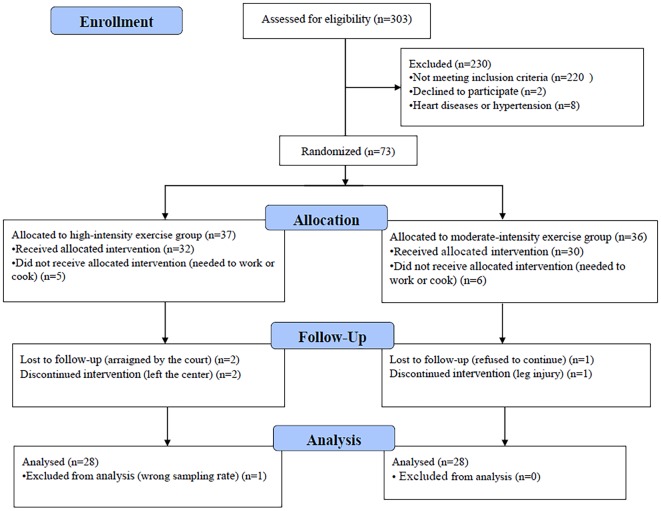
Flowchart of participant enrollment.

In total, 73 of 303 eligible participants were randomized to either the moderate- or high-intensity aerobic exercise group; 56 participants completed the entire trial (Figure 1). One participant’s data were excluded from the analysis because the wrong fNIRS data acquisition sample rate was used; the standard sample rate was 7.81 Hz, whereas the excluded participant’s sample rate was 3.91 Hz. There were no significant differences between the moderate-intensity (*n* = 28) and high-intensity (*n* = 28) exercise groups in demographic characteristics (age, weight, and height), fitness (BMI and resting heart rate), or drug use (duration, usage, and frequency) prior to exercise intervention (Table 1). Each group completed testing at baseline and after exercise and rest, with 1 week separating the exercise and rest test sessions, and in a counterbalanced order. During the preliminary session, anthropometric data were collected, and participants were asked not to engage in strenuous exercise or to drink alcohol for 24 h prior to testing. Eating or drinking of caloric or caffeinated beverages was to be avoided 2 h prior to testing.

**Table 1:** Demographic and fitness characteristics of all participants by exercise intensity.

	Total		Moderate intensity		High intensity		*t*-test score
Characteristic	Mean	SD	Mean	SD	Mean	SD	
Demographic						
Age (year)	31.1	4.4	30.5	3.3	31.8	5.2	0.26
Height (m)	1.7	0.1	1.7	0.1	1.7	0.1	0.90
Weight (kg)	72	11.3	72.5	12.1	71.5	10.5	0.74
Fitness						
BMI (kg/m^2^)	24.7	3.5	24.9	3.8	24.6	3.2	0.75
Resting heart rate (bpm)	74.3	7.6	73.6	7.1	75	8.3	0.55
Methamphetamine use						
Duration (year)	6.4	2.8	6.0	3.1	6.8	2.5	0.27
Usage (g/dose)	0.3	0.3	0.3	0.3	0.3	0.3	0.72
Frequency (days/week)	2.8	2.3	2.6	2.2	3.0	2.5	0.53

### Visual Food Cue Paradigm

**Figure 2:**
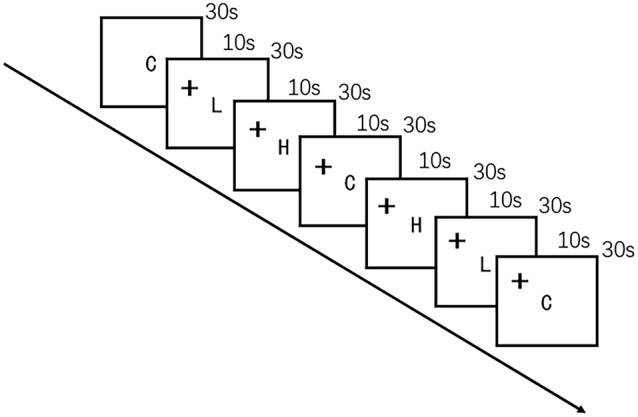
Schematic representation of the visual food cue paradigm procedure. C indicates control; L, low-calorie food; H, high-calorie food; and +, fixation cross.

The food cue paradigm was adapted from a previous study (Killgore et al., 2003) by using high-quality, full-color photographs from a commercial stock photography website^1^. Participants were instructed to look carefully at each image while undergoing fNIRS. The images were presented on a computer screen positioned 30 cm from the eyes of the participant. Control images were of non-food objects from nature (such as flowers) that were similar in shape, color, and texture. Low-calorie food images were of fruits and vegetables, whole-grain foods, and the like, whereas high-calorie food images were of hamburgers, ice cream, etc. Seven alternating blocks of 10 images of food and non-food images each were shown for 3 s per image in the order presented in Figure 2. The blocks were delimited by a 10-s fixation cross (+). The entire scan lasted 240 s.

### fNIRS Data Acquisition

**Figure 3:**
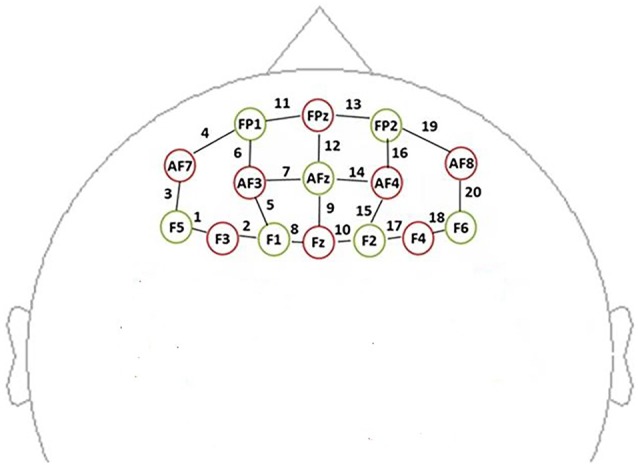
Schematic showing the near-infrared spectroscopy (NIRS) probe array arrangement (front view). The NIRS probe comprises eight sources (red) and seven detectors (yellow). We defined the channel location as the midpoint of the source-detector distances, labeled 1–20.

In this study, data were recorded by using a multichannel, continuous wave, fNIRS instrument (NIRScout, NIRx Medical Technologies LLC; Minneapolis, MN, USA). We acquire dual-wavelength (760 and 850 nm) near-infrared light to measure the relative concentration changes in oxyHb and deoxyHb (Maki et al., 1995; Yamashita et al., 1996) based on the modified Beer-Lambert law (Cope et al., 1988) with a sampling frequency of 7.81 Hz. For the NIRS experiment, eight sources and seven detectors (yielding 20 channels) were placed over the PFC region (see Figure 3). Sensors were located by aligning the bottom row of electrodes with the International 10–20 sites AF7-Fp1-Fpz-Fp2-AF8 line (Jurcak et al., 2007). The distance between the source and the detector was 3 cm. The midpoint of the source geophone distance was defined as the channel position. Motion artifacts were maintained at a minimum by asking the participants to remain still during the probe.

### fNIRS Data Processing

The fNIRS data were assessed with Homer2 software (MGH-Martinos Center for Biomedical Imaging, Boston, MA, USA) using Matlab (Mathworks, Natick, MA, USA). Motion artifacts were detected within 0.5 s when the signal changed by more than 10% of the SD. Wavelet filtering was used in Homer2 to detect and remove these artifacts (Molavi and Dumont, 2010). The fNIRS signals were preprocessed: baseline drift was removed using a high-pass filter with a cut-off frequency of 0.01 Hz, and a low-pass filter with a frequency of 0.1 Hz was used to reduce the influence on the signal of heartbeat, respiration, blood pressure, and skin blood flow. Hemoglobin concentration changes were calculated using the modified Beer-Lambert law. Block averaging was performed on the data to obtain the average response of each participant to the images at the 20 channels before and after exercise. The magnitude of change in the HbO concentration was used as the primary measure because it has a better signal-to-noise ratio than does HbR (Strangman et al., 2002).

### Subjective Appetite Sensations

Subjective appetite sensations were measured immediately after exercise or rest using visual analog scales on an electronic appetite self-rating system (Gibbons et al., 2011). Participants rated the following three feelings using a visual analog scale that ranged from “not at all” to “extremely”: their desire to eat, how full they felt, and how hungry they felt.

### Exercise Protocol

The aerobic exercise was performed using a bicycle ergometer (SH-5000U) at 50 rpm. Participants were allowed to warm-up for 5 min and to cool down for 5 min. The experimental exercise period was 25 min. For this period, the participant’s heart rate was maintained either within 65%–75% or within 76%–85% of their estimated maximum heart rate (calculated as 206.9–0.67 × age; Gellish et al., 2007). Heart rate was monitored using a Suunto Smart Sensor (Suunto Oy, Vantaa, Finland). The participants in the control rest condition performed no exercise. Instead, they sat in a quiet room for 35 min and read about drug use disorder treatments as well as exercise- and fitness-related material.

### Statistical Analysis

Statistical analyses were performed using IBM SPSS for Windows (Chicago, Illinois, version 20). Two-way repeated-measures analysis of variance was used to determine the main effects of exercise intervention (control and exercise) and exercise intensity (moderate and high) and their interaction effects on the PFC region activity and appetite measures. *Post hoc* tests with Bonferroni adjustments were used to determine where significant differences existed. We corrected for multiple comparisons using the Benjamini-Hochberg false discovery rate procedure (Benjamini and Hochberg, 1995). Values are presented as means ± SDs unless otherwise stated. Differences with 2-sided *P*-values < 0.05 were considered statistically significant.

## Results

### fNIRS

**Figure 4:**
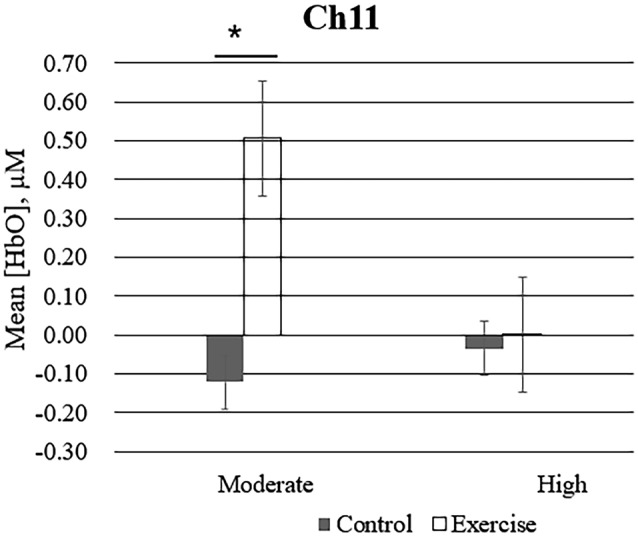
Mean changes in oxyhemoglobin concentration (HbO) for channel 11 (Ch11) as participants in the exercise and control groups view images of high-calorie foods grouped by exercise intensity. Bars represent participant means, and vertical lines represent standard errors of the mean; *P = 0.02.

Repeated measures analysis of variance results revealed significant main effects or interactions on the HbO concentration in 3 of 20 channels while participants were viewing high-calorie or low-calorie food images (Table 2). After applying *post hoc* tests and controlling for multiple comparisons using the Benjamini-Hochberg false discovery rate procedure (Benjamini and Hochberg, 1995), one channel (Ch11) remained significant, and this channel was located over the left OFC (Table 3, Figure 4). The grand averaged waveform of statistically significant HbO concentration changes in Ch11 is shown in Figure 4. There was a significant main effect of exercise (*F*_(1,54)_ = 6.66, *P* = 0.01) and of intensity (*F*_(1,54)_ = 4.34, *P* = 0.04), and a significant interaction (*F*_(1,54)_ = 5.33, *P* = 0.03) between exercise and intensity for Ch11. Furthermore, the HbO concentration changes increased significantly after moderate-intensity exercise (*P* = 0.02) but not high-intensity exercise (Figure 4). We also conducted additional analyses incorporating the BMI data as a covariate to determine whether BMI would independently affect the results. We found that the results of the analyses were similar to that of the previous analysis in that there was also a significant main effect of intensity (*F*_(1,54)_ = 4.34, *P* = 0.04) and a significant interaction between exercise and intensity for Ch11 (*F*_(1,54)_ = 5.19, *P* = 0.03). The HbO concentration changes also increased significantly after moderate-intensity exercise (*P* = 0.02).

**Table 2:** Main effects and interactions of viewing high-calorie and low-calorie food images separately in significant channels.

Channel	Moderate intensity, (HbO) change		High intensity, (HbO) change				
	Mean	SD	Mean	SD	Intensity effect	Exercise effect	Exercise × Intensity
High-calorie food images
2
Control	−0.049	0.247	−0.005	0.293	*F*_(1,54)_ = 8.02	*F*_(1,54)_ = 3.40	*F*_(1,54)_ = 3.30
Exercise	−0.046	0.409	0.286	0.506	*P* = 0.007	*P* = 0.071	*P* = 0.075
11
Control	−0.121	0.378	−0.034	0.334	*F*_(1,54)_ = 4.34	*F*_(1,54)_ = 6.66	*F*_(1,54)_ = 5.33
Exercise	0.507	0.926	0.0004	0.571	*P* = 0.042	*P* = 0.013	*P* = 0.025
Low-calorie food images
3
Control	0.009	0.285	0.126	0.279	*F*_(1,54)_ = 5.24	*F*_(1,54)_ = 0.29	*F*_(1,54)_ = 0.17
Exercise	−0.053	0.464	0.117	0.257	*P* = 0.026	*P* = 0.60	*P* = 0.69

*(HbO) indicates a relative concentration change in oxyhemoglobin*.

**Table 3:** Mean changes in oxyhemoglobin concentration (HbO) among individuals viewing high-calorie food following moderate-intensity exercise or resting control sessions measured in 20 prefrontal NIRS channels divided into four areas.

		Control, (HbO) change		Exercise, (HbO) change			
Area	Channel	Mean	SD	Mean	SD	Individual *P*-value	Corrected *P*-value^a^
OFC
	4	−0.0565	0.3818	0.3158	0.7147	0.022*	0.190
	11	−0.1207	0.3784	0.5067	0.9256	0.001*	0.020*
	13	−0.0733	0.3229	0.2900	0.6926	0.031*	0.190
	19	−0.0727	0.3512	0.1588	0.4601	0.056	0.190
VLPFC
	1	−0.0413	0.3252	−0.2063	0.7287	0.202	0.577
	3	−0.0467	0.2909	0.1715	0.4557	0.057	0.190
	18	−0.0683	0.2307	−0.0283	0.3979	0.807	0.944
	20	−0.0456	0.2062	0.1615	0.4455	0.054	0.190
DLPFC
	2	−0.0485	0.2471	−0.0463	0.4085	0.984	1.000
	5	−0.0281	0.1868	−0.0281	0.2833	1.000	1.000
	8	−0.0080	0.2582	0.0100	0.3607	0.850	0.944
	9	−0.0159	0.2722	0.0322	0.3624	0.682	0.880
	10	−0.0012	0.2490	−0.0458	0.4638	0.704	0.880
	15	0.0004	0.1625	−0.0515	0.5708	0.701	0.880
	17	−0.1252	0.4629	−0.2196	0.4953	0.646	0.880
FPA
	6	−0.0242	0.2168	0.0992	0.4313	0.231	0.578
	7	−0.0215	0.1692	0.0193	0.2524	0.516	0.880
	12	−0.0168	0.2026	0.0448	0.2736	0.473	0.880
	14	0.0085	0.1635	−0.0408	0.4784	0.665	0.880
	16	−0.0563	0.2633	0.1326	0.9148	0.370	0.822

^a^One P-value was corrected for multiple comparisons using the Benjamini-Hochberg false discovery rate procedure. DLPFC indicates, dorsolateral prefrontal cortex; FPA, frontopolar cortex; NIRS, near-infrared spectroscopy; OFC, orbitofrontal cortex; VLPFC, ventrolateral prefrontal cortex; SD, standard deviation; *P < 0.05.

### Subjective Sensations of Appetite

**Figure 5:**
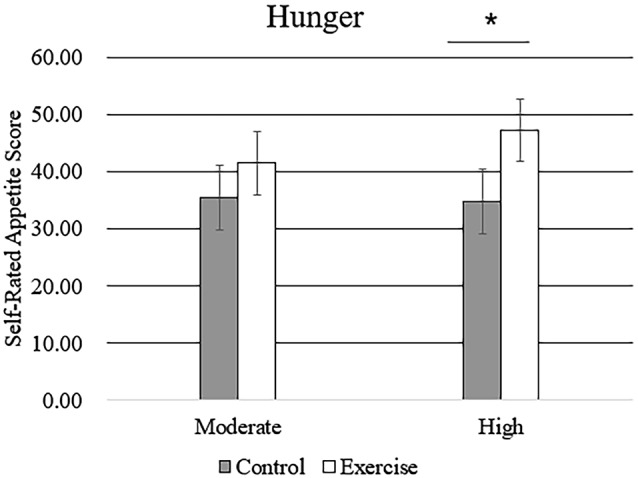
Subjective feeling of hunger after the rest period and moderate- or high-intensity exercise. Bars represent participant means; vertical lines, standard errors of the mean; *P < 0.05.

Subjective feelings of appetite, in terms of hunger, fullness, and desire to eat after exercise and control sessions, are given in Table 4. There was a higher mean rating of hunger after high-intensity exercise compared with that after moderate-intensity exercise (Figure 5), but no main effect of exercise intensity nor any significant interaction effect was found. No significant effect of exercise, intensity, or their interaction was observed for the subjective feelings of fullness or the desire to eat.

**Table 4:** Fasting subjective appetite sensations after high- or moderate-intensity exercise and resting control sessions.

	Moderate intensity, Appetite score		High intensity, appetite score				
Appetite sensation	Mean	SD	Mean	SD	Intensity effect	Exercise effect	Exercise × Intensity effect
Desire
Control	46.65	29.23	46.58	37.24	*F*_(1,54)_ = 0.06	*F*_(1,54)_ = 0.22	*F*_(1,54)_ = 0.20
Exercise	50.33	27.15	46.68	29.43	*P* = 0.808	*P* = 0.643	*P* = 0.661
Fullness
Control	55.85	27.13	50.14	29.22	*F*_(1,54)_ = 0.58	*F*_(1,54)_ = 0.02	*F*_(1,54)_ = 0.35
Exercise	53.14	24.20	51.79	24.43	*P* = 0.577	*P* = 0.888	*P* = 0.560
Hunger
Control	35.50	25.13	34.70	32.39	*F*_(1,54)_ = 0.12	*F*_(1,54)_ = 7.16	*F*_(1,54)_ = 0.91
Exercise	41.48	27.29	47.29	29.05	*P* = 0.727	***P* = 0.010**	*P* = 0.346

*The significance of bold values is 0.013*.

## Discussion

This study used fNIRS to examine the short-term effects of moderate- and high-intensity aerobic exercise on prefrontal brain activity and visual analog scale scores to assess the effects on appetite in individuals with MA dependence. The findings showed that activation of the OFC associated with viewing images of high-calorie foods increased in these individuals following moderate-intensity aerobic exercise compared with that at rest and that the subjective feeling of hunger increased in these individuals after exercise, especially after high-intensity exercise.

Thus, consistent with our hypothesis, the present study found that short-term aerobic exercise increased OFC activation to high-calorie food cues in users of MA. Studies have found that individuals who are not drug users and are either normal weight or have obesity show different neural responses to visual food cues in the reward region; people with obesity have greater neural responses to food cues than people with normal weight, no matter whether they are hungry or full (Dimitropoulos et al., 2012). This is in contrast to individuals who are drug users because people who are dependent on drugs show lower neural responses to natural reward cues than non-drug users, including to food cues (Volkow et al., 2010). Numerous studies have shown that short-term exercise can modulate appetite regulation in peripheral and central areas in normal weight, overweight, and individuals with obesity (Killgore et al., 2013; Crabtree et al., 2014). With the use of functional MRI analyses, researchers have shown that compared with controls, participants performing short-term moderate aerobic exercise show decreased activation of the insula or OFC, which are associated with hedonic “liking,” and decreased activation of the putamen, which is associated with motivational “wanting,” in response to visual food cues (Evero et al., 2012), which is inconsistent with our findings that exercise increased neuronal activation to high-calorie food cues in users of MA.

The present study showed that activation of the OFC associated with viewing images of high-calorie vs. low-calorie foods increased in these individuals following moderate-intensity aerobic exercise. This result is consistent with the finding that brain responses were positively associated with the self-rated desire to consume high-calorie food, in particular, savory food rather than sweet food. Our results also indicated that physical exercise was associated with the responsiveness of specific brain regions, and these brain region responses have been associated with the preference and desire for high-calorie foods (Killgore et al., 2013). Thus, the food images used in the present study were classified based on calorie content, not on sugar content.

The present study using neuroimaging is an early study of food reward among patients who are MA-dependent. By contrast, other such studies of human addiction have generally used healthy participants and focused on their neural responses to drug-related cues. Such studies have shown that drug-related cues activate the brain regions that are normally stimulated by nondrug-related rewards (Sell et al., 2000; Heinz et al., 2004; Wrase et al., 2007; Diekhof et al., 2008). Fewer researchers have explored brain region responses to nondrug-related rewards in persons with substance dependence on cocaine, alcohol, nicotine, opiate, or methamphetamine, and those studies have shown decreased neural responses to nondrug-related rewards. Compared with that for drug-related cues, the response of the brain is less sensitive to films of outdoor nature scenes or to explicit sexual content among cocaine users than among persons who do not use drugs (Garavan et al., 2000). People with cocaine (Goldstein et al., 2007), alcohol (Wrase et al., 2007), opiate (Martin-Soelch et al., 2010), or nicotine (Bühler et al., 2010) misuse show deficits in brain activity to monetary rewards compared with that in the control group. Such findings suggest that a lower response to reward anticipation in the ventral striatum may be a vulnerability factor for the development of early nicotine use (Peters et al., 2011). One study finds that, compared with controls, MA users chose to view more MA-related images than pleasant images, and that the lower the dopamine D2 receptor availability is in the lateral OFC, the more they chose the MA-related images, refining the central hypothesis that dopamine-system deficits contribute to drug-biased decision-making in addiction, and showing a role for the OFC (Moeller et al., 2018). The higher neural activation in the food reward region following exercise in the present study illustrates that exercise may restore the pathway hijacked by drug reward and shows activation to natural rewards similar to that among individuals without substance dependence, which is one of the signs of reward function recovery in drug users.

Previous research suggests that the prefrontal control system may be the key to successful drug withdrawal. The frontal lobe, which has been shown to modulate processes associated with reward in the striatum, is one of the regions that predict treatment outcomes and has shown increased reward and cognitive control in patients who successfully remain abstinent (Garavan and Weierstall, 2012). Although the present experiment monitored the activation of the PFC regions only through fNIRS, the PFC has a strong relationship with both drug dependence and food reward. The present study showed that aerobic exercise increased the activation of the OFC to the presentation of images of high-calorie foods, which is consistent with studies in primates that have shown that the amygdala and the ventral PFC, especially the orbitofrontal regions, are important to the visual evaluation of food stimuli (Rolls, 1984, 1990; Wilson and Rolls, 1993). In addition, decreased activity in the OFC is consistent with a decreased decision-making ability and decreased food pleasantness and palatability (Zald, 2009). Therefore, the PFC, as an important brain region in drug users, can monitor the activation of drug cues and food cues on the one hand, and the executive function of drug users on the other hand, and is thus a crucial brain region associated with the recovery of cognitive function in drug users.

Effects of exercise have been investigated in substance use disorder. Research has found that 10 min of exercise significantly reduces responses to images related to smoking in the OFC and dorsolateral PFC, as well as the subjective perception of cravings, consistent with reduced activation in food reward areas and better inhibitory control (Janse Van Rensburg et al., 2009). Several studies have shown that exercise improves cognitive function while reducing drug-related craving and relapse rates. It has been shown that structured exercise training can ameliorate striatal dopamine D2/D3 receptor deficits in MA users (Robertson et al., 2016). Moderate-intensity short-term aerobic exercise has been found to reduce drug craving in persons with MA dependence and to promote drug-related inhibitory control (Wang et al., 2015, 2016). It has also been found that MA use decreases in individuals with MA dependence after exercise (Rawson et al., 2015). Combined with the results of the present study, these findings indicate that moderate aerobic exercise both reduces drug-related cravings and improves neural activation of the food reward brain region. Future studies should examine the combination of food reward and drug craving as specific indicators of reward function repair with exercise intervention among people who are dependent on drugs.

There are a number of limitations to consider in the evaluation of this study. First, our experiment was conducted in a drug rehabilitation center, with many practical and ethical restrictions regarding patient participation in studies. Therefore, it was not possible to bring people out of the center to conduct fMRI scans to measure neural activity in other brain regions. Second, The effect of an exercise intervention on food reward may vary according to an individual’s BMI (Rothemund et al., 2007; Stoeckel et al., 2008), which may have contributed to the large standard deviation in the present experimental results. Thus, future studies may be needed to confirm the present results using larger populations sizes and using BMI as a variable. Third, hunger ratings before and after the exercise/rest and for a period afterward would be stronger for the measurement of subjective appetite. Fourth, no healthy control or non-MA–dependent individuals were recruited into the trial to compare with the MA-dependent sample, and this should be addressed in future research seeking to extend these findings. Fifth, only men participated in the study; thus, the results may not be generalizable to women.

## Conclusion

The present study reports a novel finding, to our knowledge, that short-term moderate-intensity aerobic exercise may increase neuronal responses related to food reward in the PFC region among persons who are MA-dependent. Exercise increased the appetite response, especially following high-intensity exercise. This suggests that moderate exercise may reestablish the food reward pathway acutely hijacked by drugs, restore sensitivity to natural rewards, and further promote drug withdrawal among drug-dependent users. This evidence may contribute to the development of specific exercise programs for MA-dependent populations.

## Data Availability Statement

The datasets generated for this study are available on request to the corresponding author.

## Ethics Statement

The studies involving human participants were reviewed and approved by the ethics committee of the Shanghai University of Sport. The patients/participants provided their written informed consent to participate in this study.

## Author Contributions

YZ and CZ contributed to the conception and design of the study. JW and YC conducted experiments and analyzed data. YZ wrote the first draft of the manuscript. HW and XL contributed to manuscript revision. All authors read and approved the submitted version.

## Conflict of Interest

The authors declare that the research was conducted in the absence of any commercial or financial relationships that could be construed as a potential conflict of interest.

## Footnotes

^1^https://www.58pic.com/tupian/shiwutupian.html
